# Nosocomial infection control in healthcare settings: Protection against emerging infectious diseases

**DOI:** 10.1186/s40249-016-0118-9

**Published:** 2016-04-12

**Authors:** Chuanxi Fu, Shengyong Wang

**Affiliations:** Guangzhou Center for Disease Control and Prevention, Guangzhou, 510440 China; Medical College, Jinan University, Guangzhou, 510632 China

**Keywords:** Middle East respiratory syndrome (MERS), Nosocomial infection, Severe acute respiratory syndrome (SARS)

## Abstract

The Middle East respiratory syndrome (MERS) outbreak in Korea in 2015 may be attributable to poor nosocomial infection control procedures implemented. Strict infection control measures were taken in the hospital where an imported case with MERS was treated in southern China and 53 health care workers were confirmed to be MERS-CoV negative. Infection control in healthcare settings, in which patients with emerging infectious diseases such as MERS, Ebola virus disease, and the severe acute respiratory syndrome (SARS) are diagnosed and treated, are often imperfect. When it comes to emerging or unknown infectious diseases, before the imported case was finally identified or community transmission was reported, cases have often occurred in clusters in healthcare settings. Nosocomial infection control measures should be further strengthened among the workers and inpatients in designated healthcare settings that accommodate suspected cases suffering from emerging or unknown infectious diseases.

**Electronic supplementary material:**

The online version of this article (doi:10.1186/s40249-016-0118-9) contains supplementary material, which is available to authorized users.

## Multilingual abstracts

Please see Additional file 1 for translation of the abstract into five official working languages of the United Nations.

## Background

Which intervention and control measures should be immediately taken when suspected Middle East respiratory syndrome (MERS) case(s) are imported from abroad? High priority should be given to the prevention of nosocomial infections among the workers and inpatients in the healthcare settings. Since the first human case of MERS coronavirus (MERS-CoV) was reported in Saudi Arabia in 2012, 1684 laboratory-confirmed cases and 600 related deaths have been recorded (as of January, 2016) [1]. As a result, great concerns about a potential pandemic arose [2, 3]. Although the majority of cases occurred in countries in the Arabian Peninsula, international spread of MERS due to travel has been reported in at least 20 other countries, with many cases emerging in the healthcare settings [4].

On May 27, 2015, a suspected human case of MERS with a fever of 39.7 °C, who had travelled from South Korea to Huizhou city, Southern China, was notified by the World Health Organization (WHO). This suspected case was then quarantined in a negatively pressurized room and confirmed to be infected with MERS on May 29. Strict infection control measures were taken in the hospital where the case was treated to reduce the risk of further transmission. On June 10, serum samples were collected from 53 healthcare workers who used personal protective equipment when treating this patient, and all were confirmed to be MERS-CoV negative [5].

## Poor nosocomial infection control may play an important role in emerging infectious disease outbreaks, including MERS

The first imported case of MERS in Korea was reported on May 20, 2015. Over the following weeks, the number of secondary and subsequent cases from this patient increased, leading it to become the largest case cluster of MERS outside the Middle East. All cases (excluding the index case) have been linked to a single chain of transmission and were associated with healthcare facilities [6]. Of these, 30 were secondary cases, 124 were third generation cases, 24 were fourth generation cases, and 0 were fifth generation case. The index patient of the MERS outbreak in South Korea was diagnosed nine days after the onset of symptoms, and was responsible for generating the secondary cases [7]. Nearly two-thirds of the cases have been reported from St. Mary’s Hospital, Seoul, Korea, where the air-conditioning system’s lack of ventilators resulted in MERS-CoV being detected in bathrooms and on doorknobs [4]. The ineffective infection control procedures implemented during this MERS outbreak may be attributable to poor nosocomial infection control.

When it comes to emerging infectious diseases such as the severe acute respiratory syndrome (SARS), Ebola virus disease, and MERS, before the imported case was finally identified or community transmission was reported, cases have often occurred in clusters in healthcare settings. In March 2003, for example, 19 medical staff members, one inpatient, and one family number were infected with SARS-CoV by the same index SARS patient in a hospital in Guangdong [8]. In a Beijing hospital in 2003, 23 first and secondary cases were infected, of which 16 were healthcare workers, two were inpatients, and five were family numbers [9]. In the US, two nurses were infected with the Ebola virus when treating an Ebola patient [10]. A comparative study on the transmission of emerging infectious diseases in the healthcare setting showed that both SARS and MERS were found to be nosocomial super-spread at the early stage, and the reproduction number dropped below 1 within 3 to 5 generations. More SARS cases were found among healthcare workers during the outbreak, while MERS cases occurred among the patients who sought care in the same healthcare settings as the index case [7]. Infection control measures are critical in preventing the spread of infectious diseases in healthcare facilities among, for instance, medical personnel who have direct contact with patients, patients sharing an intensive care unit (ICU) room with the case patient, as well as family members visiting inpatients.

## Nosocomial infection control measures should be further strengthened in designated healthcare settings that accommodate suspected cases suffering from emerging infectious diseases

In many countries including China, nosocomial infection regulations have been established nationally, in order to ensure routine infection control operation in healthcare settings. However, in healthcare settings where patients with emerging or unknown infectious diseases are diagnosed and treated, especially those operated by local governments, infection control methods against severe infectious diseases such as MERS are often imperfect. For example, a survey of device-associated healthcare-associated infection (DA-HAI) rates in 398 ICUs in Shanghai, China, reported a high number of DA-HAIs, which posed a major threat to patient safety [11]. Therefore, great efforts are needed to strengthen and enhance the capacity of infectious disease control in local healthcare units. Equipment, facilities, supplies, and standards of operations need to be improved. More importantly, all personnel in healthcare settings, such as doctors, nurses, and administrative and other staff, have to develop their own awareness, essential knowledge, and skills in order to protect themselves against emerging infectious diseases.

Proper control measures should be taken to ensure that nosocomial infections do not occur in designated healthcare settings that accommodate suspected cases suffering from emerging infectious diseases. To this end, a comprehensive and detailed evaluation of nosocomial infection control should be conducted in each designated healthcare setting. Negative-pressure quarantine ICUs and wards should be built in accordance with the demand in the area. Regulations, standards, procedures, and operational instructions on protection against infections from emerging respiratory, gastrointestinal, body fluid, and insect-borne infectious diseases should be established. Periodical regular trainings for the knowledge of prevention and control emerging or unknown infectious diseases, and emergency exercises regarding nosocomial infection events among medics can also help to strengthen the infection control system.

## Additional file

Additional file 1: Multilingual abstracts in the five official working languages of the United Nations. (PDF 322 kb)

## Abbreviations

DA-HAI: device-associated healthcare-associated infection
ICU: intensive care unit
MERS: Middle East respiratory syndrome
MERS-CoV: Middle East respiratory syndrome coronavirus
SARS: Severe acute respiratory syndrome
WHO: World Health Organization
