# Supplementary material for: Nosocomial infection control in healthcare settings: Protection against emerging infectious diseases
Source: Infect Dis Poverty. 2016 Apr 12;5:30. doi: 10.1186/s40249-016-0118-9 (PMC4828876; doi:10.1186/s40249-016-0118-9)
Supplement: Additional file 1: — Multilingual abstracts in the five official working languages of the United Nations. (PDF 322 kb) [file 40249_2016_118_MOESM1_ESM.pdf]

Translation of the abstract into the five official working languages of the United Nations

مكافحة العدوى المكتسبة بالمستشفيات في مرافق الرعاية الصحية: الوقاية من الأمراض المعدية الناشئة

شوانشو فو وشينجيونج وانج

الخلاصة

قد يُعزى تفشّي متلازمة الشرق الأوسط التنفسية (MERS) في كوريا إلى ضعف إجراءات تنفيذ مكافحة العدوى المكتسبة بالمستشفيات. وقد اتّخذت تدابير صارمة لمكافحة العدوى في المستشفى الذي خضعت فيه أول حالة وافدة مصابة بمتلازمة الشرق الأوسط التنفسية في جنوبي الصين، في الوقت الذي تأكد عدم إصابة 53 عاملاً في مجال الرعاية الصحية بمرض متلازمة الشرق الأوسط التنفسية - فيروس كورونا. وغالبًا ما تضعف طرق مكافحة العدوى في مرافق الرعاية الصحية، والتي يخضع فيها المرضى المصابون بأمراض معدية ناشئة مثل متلازمة الشرق الأوسط التنفسية وفيروس إيبولا والمتلازمة التنفسية الحادة الوخيمة (سارس) للتشخيص والعلاج. وعندما يتعلق الأمر بالأمراض المعدية الناشئة أو غير المعروفة، قبل الانتهاء من تحديد الحالة الوافدة أو الإبلاغ عن الانتقال الجماعي للعدوى، فغالبًا ما تحدث الحالات في مجموعات بمرافق الرعاية الصحية. لذا ينبغي الزيادة من تعزيز تدابير مكافحة العدوى المكتسبة بالمستشفيات بين العاملين والمرضى بالمستشفيات في مرافق الرعاية الصحية المخصصة التي تستوعب الحالات المشتبه فيها التي تعاني من الأمراض المعدية الناشئة أو غير المعروفة.

Translated from English version into Arabic by Muhammad Said, through

TRANSLATORS
WITHOUT BORDERS

医疗机构中的医院感染控制：应对新发传染病的防护

傅传喜，王声湧

摘要

2015 年韩国暴发中东呼吸综合征（MERS）可能归结于未执行严格的医院感染控制措施。在中国南方对一例 MERS 病例治疗过程中，医院采取了严格的感染控制措施，53 名相关医护人员最终确认 MERS 冠状病毒阴性。诊治新发传染病如 MERS、埃博拉出血热和重症急性呼吸综合征（SARS）定点医疗机构的医院感染控制环节多存在不足，在新发或不明原因传染病的输入性病例最终诊断或社区暴发报告前，在医疗机构中往往已出现集中的病例。应进一步加强收治可疑新发或不明原因传染病医院的医务人员和病人中感染控制措施。

Translated from English version into Chinese by Fu Chuanxi

Contrôle des infections nosocomiales au sein d’établissements de santé : Protection contre des maladies infectieuses émergentes

Chuanxi Fu et Shengyong Wang

Résumé

L’épidémie du syndrome respiratoire du Moyen-Orient (MERS) en Corée en 2015 peut être attribuée à des

procédures de contrôle des infections nosocomiales insuffisantes. De strictes mesures de contrôle des infections ont été prises à l'hôpital du sud de la Chine ayant accueilli un cas importé atteints du MERS et où 53 personnels de santé se sont avérés être négatifs au MERS-CoV. Les méthodes de contrôle des infections au sein d'établissements de santé diagnostiquant et prenant en charge des patients atteints de maladies infectieuses émergentes, telles que le MERS, Ebola et le syndrome respiratoire aigu sévère (SRAS), sont souvent imparfaites. Les cas de maladie infectieuse émergente ou inconnue sont souvent apparus de manière groupée au sein d'établissements de santé avant l'identification finale du cas importé ou le signalement d'une transmission au sein d'une communauté. Les mesures de contrôle des infections nosocomiales doivent encore être renforcées parmi le personnel et les patients hospitalisés au sein des établissements de santé désignés qui prennent en charge des cas suspects atteints de maladie infectieuse émergente ou inconnue.

Translated from English version into French by eric ragu, through

Контроль нозокомиальной инфекции в медицинских учреждениях: профилактические мер в отношении новых инфекционных заболеваний

Чуанси Фу, Шенйонг Ванг

Краткое содержание

Вспышка ближневосточного респираторного синдрома (БВРС) в Корее в 2015 может быть связана с плохим осуществлением контроля за нозокомиальной инфекцией. В больнице на юге Китая, где был отмечен занесенный случай БРВС, были приняты жесткие контрольные меры и у 53 медицинских работников подтвержден негативный БРВС-КоВ. Методы инфекционного контроля в медицинских учреждениях, в которых находятся и проходят лечение пациенты с такими инфекционными заболеваниями, как БВРС, вирус Эбола и тяжелым острым респираторным синдромом (ТОРС), зачастую несовершенны. Когда дело касается новых и неизвестных инфекционных заболеваний, до конечного выявления или информирования общественности в медицинских учреждениях часто отмечались вспышки заболевания. Меры контроля нозокомиальной инфекции в будущем должны усиливаться как среди медицинского персонала, так и среди пациентов при выявлении случаев новых и неизвестных болезней.

Translated from English version into Russian by Jekaterina Merkuljeva, through

Control de las infecciones nosocomiales en el marco de la atención de la salud: Protección contra enfermedades infecciosas emergentes

Chuanxi Fu y Shengyong Wang

Resumen

El brote de síndrome respiratorio de Oriente Medio (MERS) en Corea en el año 2015 puede ser atribuible a la mala calidad de los procedimientos de control implementados para las infecciones nosocomiales. Se tomaron estrictas medidas de control de infección en el hospital del sur de China donde se trató un caso importado con MERS y donde se confirmó que 53 trabajadores de la salud eran MERS-COV negativos. Los métodos de control de infección en el marco de la atención de la salud, donde se diagnostica y se trata a pacientes con enfermedades infecciosas emergentes como el MERS, la enfermedad por el virus del ébola y el síndrome respiratorio agudo severo (SRAS) son a menudo imperfectos. Cuando se trata de enfermedades infecciosas emergentes o desconocidas, antes de que el caso importado sea finalmente identificado o la transmisión comunitaria reportada, por lo general se producen casos grupales en el marco de la atención de la salud. Las medidas de control de infección nosocomial deben fortalecerse aún más entre los trabajadores y los pacientes hospitalizados en ciertos entornos de atención de la salud que alojan a casos en los que se sospecha de enfermedades infecciosas emergentes o desconocidas.

Translated from English version into Spanish by Maria Alejandra Aguada, through

TRANSLATORS
WITHOUT BORDERS
